# Localization of Pyranose 2-Oxidase from *Kitasatospora aureofaciens*: A Step Closer to Elucidate a Biological Role

**DOI:** 10.3390/ijms24031975

**Published:** 2023-01-19

**Authors:** Ludovika Jessica Virginia, Clemens Peterbauer

**Affiliations:** 1 Food Biotechnology Laboratory, Department of Food Science and Technology, BOKU—University of Natural Resources and Life Sciences, Muthgasse 11, 1190 Vienna, Austria; 2 Doctoral Programme BioToP—Biomolecular Technology of Proteins, BOKU—University of Natural Resources and Life Sciences, Muthgasse 11, 1190 Vienna, Austria

**Keywords:** pyranose oxidase, *Streptomyces lividans*, Secretion, *Kitasatospora aureofaciens*, fluorescent

## Abstract

Lignin degradation in fungal systems is well characterized. Recently, a potential for lignin depolymerization and modification employing similar enzymatic activities by bacteria is increasingly recognized. The presence of genes annotated as peroxidases in Actinobacteria genomes suggests that these bacteria should contain auxiliary enzymes such as flavin-dependent carbohydrate oxidoreductases. The only auxiliary activity subfamily with significantly similar representatives in bacteria is pyranose oxidase (POx). A biological role of providing H_2_O_2_ for peroxidase activation and reduction of radical degradation products suggests an extracellular localization, which has not been established. Analysis of the genomic locus of POX from *Kitasatospora aureofaciens* (*Ka*POx), which is similar to fungal POx, revealed a start codon upstream of the originally annotated one, and the additional sequence was considered a putative Tat-signal peptide by computational analysis. We expressed *Ka*POx including this additional upstream sequence as well as fusion constructs consisting of the additional sequence, the *Ka*POx mature domain and the fluorescent protein mRFP1 in *Streptomyces lividans*. The putative signal peptide facilitated secretion of KaPOx and the fusion protein, suggesting a natural extracellular localization and supporting a potential role in providing H_2_O_2_ and reducing radical compounds derived from lignin degradation.

## 1. Introduction

Lignocellulose, a composite of the polymers lignin, cellulose, hemicellulose and pectin, provides structural integrity and resistance to pathogens and herbivores to plants. The paracristallinity of cellulose, the complexity of the hemicellulose matrix and the linkage to lignin are major barriers against the enzymatic degradation of lignocellulose [1]. The lignin fraction is a heterogenous alkyl-aromatic polymer formed from three aromatic alcohols with different degrees of methoxylation. Lignification happens through monomer and polymer crosslinking via radicals produced by oxidases and is characterized by a large number of different interunit linkages, resulting in a remarkable recalcitrance to degradation [1,2].In nature, degradation of lignocellulose is feasible at physiological conditions by a number of organisms. The process of lignin degradation is mostly described in white-rot and brown-rot fungi [3,4], which mineralize lignin through a cocktail of oxidative enzymes, namely polyphenol oxidases (laccases) and peroxidases (lignin, manganese and versatile peroxidases), as well as auxiliary enzymes such as H_2_O_2_-generating oxidases. These peroxide-providing enzymes can belong to the Glucose-Methanol-Choline (GMC) oxidoreductase family of flavoenzymes (Auxiliary Activities Family 3), such as aryl alcohol oxidases, pyranose oxidases or cellobiose dehydrogenases, or to the copper radical oxidases (Auxiliary Activities Family 5), such as glyoxal oxidase [2]. Additionally, peroxide providing enzymes may be involved in the oxidative depolymerization of cellulose and hemicellulose through lytic polysaccharide monoxygenases (LPMOs), for which hydrogen peroxide is also an essential co-substrate [5]. Most flavoprotein oxidases, such as aryl alcohol oxidases and pyranose oxidases, also have a significant dehydrogenase activity using (substituted) quinones as electron acceptors, often with a higher efficiency than molecular oxygen [6,7,8]. This dehydrogenase activity may also be involved in lignin depolymerization, as has been shown in vitro for combinations of fungal laccase and pyranose oxidase [9] as well as lignin peroxidase and pyranose oxidase [6,10], where addition of pyranose oxidase increased the degree of depolymerization markedly, presumably by reducing the resulting lignin breakdown products and preventing their immediate repolymerization.

While the enzymatic system(s) for depolymerization of lignin in fungi are well investigated, this is not the case for bacteria, although a potential for lignin depolymerization and modification using similar enzymatic activities such as laccases and peroxidases is increasingly recognized [1,11,12,13]. To date, there are only few reports on flavoprotein oxidoreductases that can be considered Auxiliary Activities for lignocellulose degradation. Pyranose oxidases were characterized from *Pseudarthrobacter siccitolerans* [14], *Kitasatospora aureofaciens* [6] and *Streptomyces canus* [15]. The sequence of POx derived from *K. aureofaciens* (*Ka*POx) is closely related to fungal POx [16] and shows both oxidase and dehydrogenase activity with comparable substrate preferences to fungal enzymes. The oxidase activity of POx is suitable for providing H_2_O_2_ for peroxidase activation for lignin depolymerization, while the dehydrogenase activity can play a role in preventing re-polymerization of lignin-derived radicals as well as protecting the cells from damage by those radicals. Both putative biological roles are only plausible if POx is located extracellularly, otherwise the produced hydrogen peroxide would have to be transported out of the cells for activation of peroxidases, and free radicals derived from lignin depolymerization would have to be imported into the cells. Both bacterial genes were identified by sequence similarities and were expressed in *E. coli* for characterization, but their natural subcellular localization has not been established. It should be noted that a copper radical oxidase with catalytic similarities to glyoxal oxidase (for which similar biological functions are discussed) has been described as a secretory enzyme in *Streptomyces coelicolor* and *Streptomyces lividans* [17]. We investigated the subcellular location of *Ka*POx by heterologous expression using Gram-positive bacterial expression systems and included additional sequences upstream of the annotated ATG that may constitute a functional signal peptide. To accommodate the detection of secreted enzyme, fusion constructs with a fluorescent protein (mono-Red Fluorescent Protein, mRFP) were used as reporter.

## 2. Results

### 2.1. Signal Peptide Prediction

**Figure 1:**
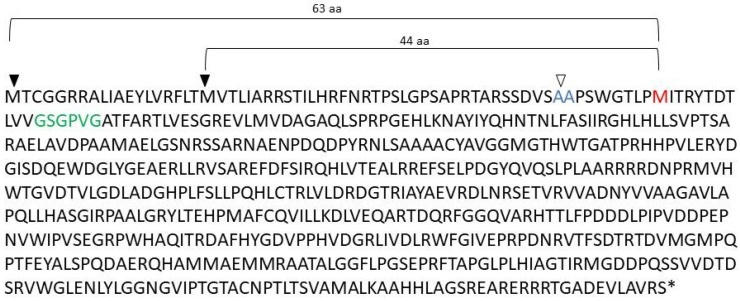
Results from ORF Finder showing two Methionine (M) in frame in the upstream region (filled triangles), followed by the previously annotated start codon (M in red). The putative cleavage site is shown in blue with an open triangle. The Rossman fold motif is shown in green.

The *K. aureofaciens* genomic sequence upstream of the coding region of *Ka*POx was analyzed using ORF Finder: https://www.ncbi.nlm.nih.gov/orffinder/ (accessed on 11 November 2019), and two Met residues were discovered upstream and in frame with the previously annotated start codon (Figure 1). These additional sequences amount to 63 amino acids starting from the first and 44 amino acids starting from the second encoded Met residue. These sequences were analyzed using TatP-1.0 [18]: https://services.healthtech.dtu.dk/service.php?TatP-1.0 (accessed on 11 November 2019). The longer sequence comprising 63 AA was classified as unlikely to be a Tat signal peptide, but part of the 44 AA sequence was found to score above the cut-off in all but one category. The potential signal peptide cleavage site was predicted to be between positions 36 and 37 (Supplemental Material, Figure S1). 

### 2.2. Heterologous Expression of KaPOx with a Putative Signal Peptide

**Figure 2:**
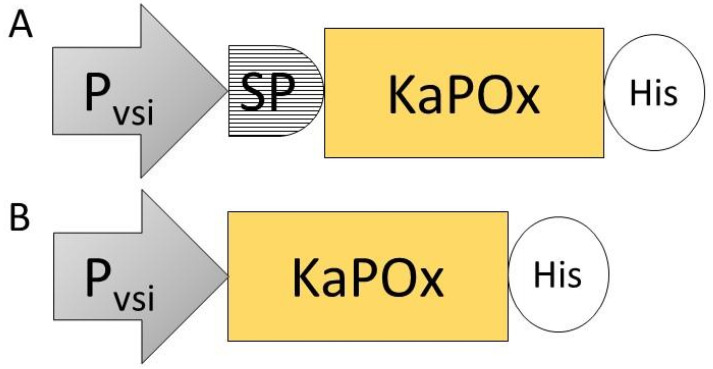
Expression constructs SP*Ka*POxHis (**A**) and *Ka*POxHis (**B**), containing the constitutive promoter of the *S. venezuelae* subtilisin inhibitor gene (P_vsi_), the putative signal peptide (SP) identified upstream of the mature domain of pyranose oxidase from *K. aureofaciens* (KaPOx) and a C-terminal 6× His-tag (His).

**Figure 3:**
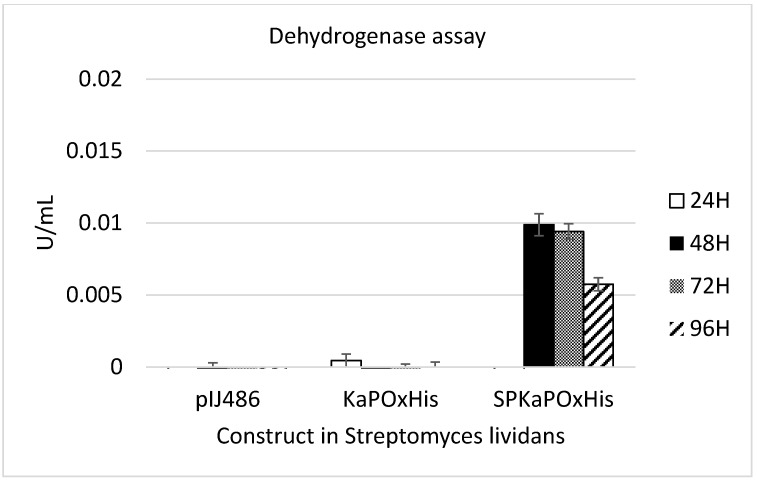
Dehydrogenase activity assay of *S. lividans* supernatant obtained from cultures harboring the empty vector pIJ486, pIJ486-*Ka*POxHis, and pIJ486-SP*Ka*POxHis. Samples were taken from the supernatant at 24, 48, 72 and 96 h (H) as indicated.

To investigate whether the 44 AA upstream of the originally annotated ATG of *Ka*POx constitute a functional signal peptide as predicted, constructs with and without this additional sequence (SP*Ka*POxHis and KaPOxHis, respectively) were prepared and heterologously expressed as described (Figure 2). Expression in *B. subtilis* did not result in detectable levels of secreted protein with either construct, but the His-tagged protein was detectable in the cell extract by Western blotting (Supplementary Figure S2). Protein purification by affinity chromatography was possible from the cell extract, but not from the supernatant. Yields of purified protein were considerably lower from the extract of cultures expressing the SP*Ka*POxHis construct (Figure S2). Expression in *S. lividans* was carried out using the constitutive promoter P_vsi_ and pIJ486 as plasmid backbone (Figure 2). Secreted *Ka*POx could be detected in the supernatant of *S. lividans* carrying the construct with the putative SP by enzymatic assay at a volumetric activity of 0.01 U/mL (dehydrogenase activity; Figure 3) as early as 48 h after start of the cultivation. 

### 2.3. Fusion Protein Approach

**Figure 4:**
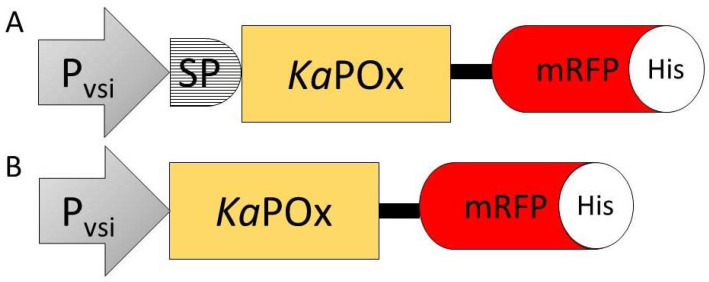
Constructs SP*Ka*POxmRFPHis (**A**) and *Ka*POxmRFPHis (**B**) containing the constitutive *S. venezuelae* P_vsi_ promoter (P_vsi_), the putative signal peptide (SP), the *Ka*POx mature domain, the mRFP fluorescent protein (mRFP) and a C-terminal 6×His-Tag (His).

Translational fusions of the fluorescent mono-Red Fluorescent Protein (mRFP) C-terminally of *Ka*POx with a 6× His-Tags added C-terminally of mRFP were constructed (Figure 4). 

**Figure 5:**
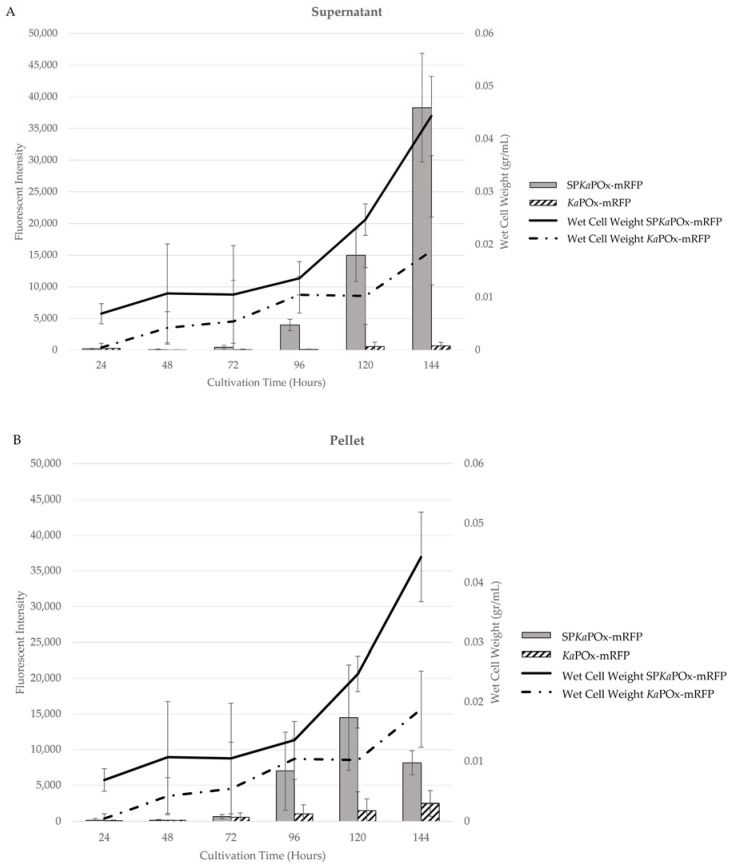
Growth curve (in wet cell weight) and mRFP fluorescence intensity from cultures expressing the fusion constructs SP*Ka*POxmRFPHis and *Ka*POxmRFPHis in the supernatants (**A**) and the pellet fractions (**B**). Fluorescent intensity values are adjusted for the recorded wet cell weight and are the mean results of three cultures.

Both fusion constructs (SP*Ka*POxmRFPHis and *Ka*POxmRFPHis) were expressed in *S. lividans* under the control of the P_vsi_ promoter. In the culture supernatants, SP*Ka*POxmRFPHis showed a very low level of fluorescence at 72 h that increased constantly in intensity until 144 h. The supernatant from *Ka*POxmRFPHis showed a much lower fluorescence, detectable only at 120 and 144 h (Figure 5). 

Fluorescence was also detected in the cell pellets starting at 72 h in cultures of both constructs. Intensity increased until the end of cultivation at a very low level for cultures harboring *Ka*POxmRFPHis; for cultures harboring SP*Ka*POxmRFPHis a stronger increase was measured until 120 h, followed by a decline at 144 h. Wet cell weight (WCW) was monitored over time, and the obtained values were used to normalize the measured fluorescence intensity. A different growth behavior for cells harboring the different constructs was observed: cultures harboring *Ka*POxmRFPHis showed a constant but slow increase in wet cell weight, with a final value after 144 h, approximately half of that recorded for cultures of SP*Ka*POxmRFPHis, in which a marked increase after 96 h was observable. 

**Figure 6:**
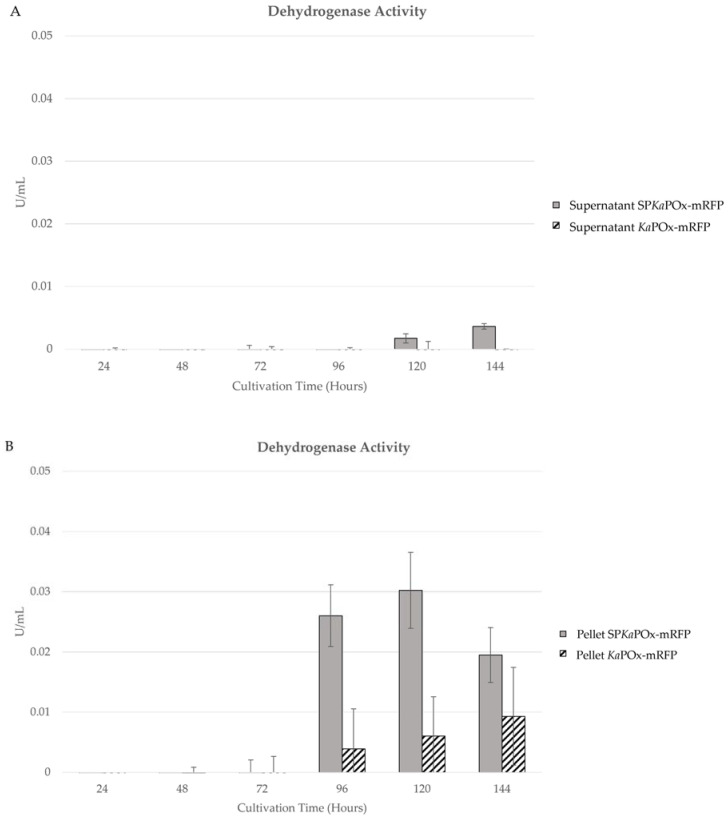
Enzymatic activity (dehydrogenase) in samples of the supernatant (**A**) and the pellet fractions (**B**) of cultures harboring SP*Ka*POxmRFPHis and *Ka*POxmRFPHis. Activity values are normalized for the recorded wet cell weight, as in Figure 5.

Enzymatic activity assays were also done on the samples from supernatants and pellets of cultures harboring both constructs. Both oxidase as well as dehydrogenase activity could be detected in the pellets starting at 96 h (oxidase activity is not shown, as the values were consistently very low). In the supernatants, no activity was measurable for cultures of *Ka*POxmRFPHis. Cultures containing SP*Ka*POxmRFPHis showed low activity at 120 and 144 h (Figure 6A). Enzymatic activities in the cell pellet increased slowly on a low level for *Ka*POxmRFPHis cultures. In SP*Ka*POxmRFPHis cultures, activity was higher at 96 and 120 h and declined in the last sample at 144 h. It is notable that the measured activities in the pellet fractions were higher in all corresponding samples than those in the supernatants.

**Figure 7:**
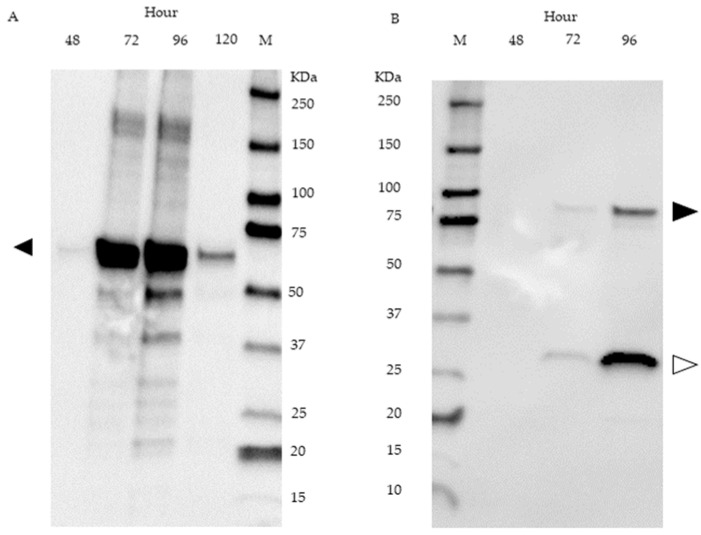
Western blot of supernatant samples from *S. lividans* cultures harboring SP*Ka*POxHis at 48, 72, 96 and 120 h (**A**) and SP*Ka*POxmRFPHis at 48, 72 and 96 h (**B**). A size marker is shown in lane M. The expected molecular weight of intact fusion protein and of the mRFP domain is 86.5 KDa (black arrow) and 26.6 KDa (white arrow), respectively.

The presence of fusion protein was detected in supernatant samples of SP*Ka*POxmRFPHis cultures by Western blot using an anti-His-tag antibody. A band corresponding to intact fusion protein is visible in the samples obtained after 72 h and after 96 h of cultivation. In both samples, a notably more intense band corresponding to a molecular weight of 27 kDa is present (Figure 7B). Western blot analysis of SP*Ka*POxHis derived from the supernatant is shown in Figure 7A.

## 3. Discussion

Here we expressed pyranose oxidase from *K. aureofaciens* (*Ka*POx), a bacterial enzyme from the family Auxiliary Activities 3 (AA3), in the two Gram-positive bacterial expression systems *B. subtilis* and *S. lividans*. We used constructs containing *Ka*POx, as previously expressed intracellularly in *E. coli* and characterized [6], as well as constructs containing the genomic sequence encoding additional 44 amino acids upstream of and in frame with the previously characterized coding sequence and also starting with an ATG. This upstream sequence was tentatively classified to constitute a Tat signal peptide by computational tools. We could not detect secretory enzyme in cultivations of both constructs expressed in *B. subtilis* but could purify the tagged protein from the cell extracts of both cultures. When using *S. lividans* as expression host, secretory enzyme with dehydrogenase activity towards glucose as the electron donor and ferrocenium hexafluorophosphate as the electron acceptor was detected in cultures harboring the construct with the putative signal peptide. The volumetric dehydrogenase activity was low at 0.01 U/mL, and oxidase activity could not be detected. Herzog et al. (2019) showed a 6.6-fold dehydrogenase activity with ferrocenium hexafluorophosphate compared to oxidase activity with molecular oxygen as electron acceptor for recombinant *Ka*POx produced in *E. coli*. We conclude that, considering the low dehydrogenase activity of secreted *Ka*POx produced in *S. lividans*, oxidase activity is probably below the detection limit.

We subsequently prepared fusion constructs with the gene encoding the fluorescent reporter protein mRFP downstream of the *Ka*POx-encoding gene (with and without the upstream sequence encoding the putative SP). Fluorescence could be detected in the supernatant after 72 h in cultures expressing the fusion construct with the putative SP, increasing significantly until 144 h and always higher than the intensity in the cell pellet except very early after initial detection (Figure 5A). When constructs without the putative SP were used, a fluorescent signal was detected in the pellet at 72 h, but only another 48 h later in the supernatant, where the intensity remained very low until the last sampling point, and significantly lower than in the cell pellets at all time points (Figure 5B). It is notable that the fluorescence intensity in the cell pellets of SP*Ka*POxmRFPHis cultures reached a peak at 120 h and decreased for the last sampling point at 144 h. Also notable is the different growth behavior of the cultures expressing the two different constructs: the wet cell weight of SP*Ka*POxmRFPHis and *Ka*POxmRFPHis cultures was comparable until 96 h, but increased markedly slower after this in *Ka*POxmRFPHis cultures to a maximal value of 0.08 g/mL compared to SP*Ka*POxmRFPHis cultures, which reached 0.140 g/mL. This is concomitant with the appearance of fluorescent fusion protein in significant amounts in the supernatant. These observations suggest that, while (over)expression of both fusion constructs constitutes a metabolic burden for the cells, secretion driven by the signal peptide (and observable in the fluorescent intensity) avoids or alleviates cellular stress through cytoplasmic accumulation of the heterologous fusion protein, resulting in healthier growth and higher biomass formation than in the cells producing fusion protein lacking the putative signal peptide, where the expressed fusion protein accumulates throughout growth. 

During six days or 144 h of cultivation, increase of wet cell weight as well as fluorescence intensity relative to WCW increased gradually without fluctuations or abrupt changes. When monitored for longer periods (until 192 h, not shown) an onset of “plateauing” of secretory fluorescence is observable in SP*Ka*POxmRFPHis cultures, while wet cell weight continues to increase for all cultures and only starts to show a lower increase rate at the last sampling point. This suggests that the cells had not yet reached the stationary phase. Since no sudden increases in extracellular fluorescence with concomitant stagnation or loss of wet cell weight were observed, we conclude that the extracellular fluorescence is a consequence of secretion, not cell death and lysis.

It is notable that the fluorescent signal from the fusion constructs was detectable later than the enzymatic activity in the previous experiments (72 h vs. 48 h). The fusion proteins are larger at 86.5 KDa than the *Ka*POx mature domain (59.9 KDa). A late secretion for heterologous enzymes in *S. lividans* was reported by Sianidis et al. (2006) [19] for the 95 KDa xyloglucanase, where secretion peaked at ~120H of cultivation time. It appears plausible that *Ka*POx is secreted earlier compared to fusion proteins containing the fluorescent reporter. 

We also measured enzymatic activity (dehydrogenase) in both cell pellets and supernatants of SP*Ka*POxmRFPHis and *Ka*POxmRFPHis cultures and observed a marked discrepancy to the results of the fluorescence measurements. *Ka*POxmRFPHis cultures followed the same pattern as observed previously, with accumulation of active enzyme in the pellet fraction and essentially no detectable activity in the supernatants. SP*Ka*POxmRFPHis cultures also showed intracellular accumulation of activity, peaking at 120 h, as did the fluorescence measurement. In the supernatants, however, the activities, while following the same pattern as in the secretory fluorescence (constant increase until 144 h), remained much lower than the measured intracellular activities. Taken by itself, this appears to argue against secretion of active enzymes to meaningful levels. Western blot analysis of supernatant samples revealed intact fusion protein in the supernatant, faintly in samples taken after 72 h and more prominently in samples taken after 96 h. At both time points, a more intense band corresponding to the molecular weight of mRFP (± 27 KDa) is also detected (Figure 7B). We conclude that the lower band represents the product of proteolytic cleavage of secreted fusion protein, namely mRFP, which is detected via the attached His-tag (the rest of the protein, i.e., the *Ka*POx mature domain, is not detectable, as it does not contain a His-tag). Since mRFP is a small, compact protein composed mostly of β-sheets, it is conceivable that it is released from the fusion protein by extracellular proteases, but stays otherwise intact and fluorescent (thus detectable in the fluorescence measurements), whereas the larger oxidoreductase domain of the fusion protein may be further degraded and rendered inactive. In this case, we can presumably detect all of the secretory fluorescence, but only a fraction of the activity. The appearance of the His-tagged mRFP domain in the supernatant by another pathway, namely cell lysis and subsequent proteolysis, or intracellular proteolysis followed by cell lysis and release, is not plausible at these time points, as this would have been obvious in other parameters. According to the structural model of *Ka*POx [6], the C-terminus is exposed on the surface of the Rossman domain, facing away from the active site as well as the dimerization interface. An interference of the mRFP domain with dimerization and/or activity appears therefore unlikely, but cannot be entirely ruled out. 

Regarding the failure to detect enzymatic activity (either kind) in the experiments with *B. subtilis*, it has to be noted that the used strain NZ8901 is not optimized for extracellular protease activity [20], which is known to be a major detrimental factor for the expression of heterologous proteins in this organism [21,22]. Since these early experiments were only done with SP*Ka*POxHis- and *Ka*POxHis-constructs, which do not allow fluorescent detection (and do not provide a more stable mRFP-domain that can be detected via the His-tag), it is conceivable that secretion driven by the putative signal peptide did, in fact, happen, but remained undetectable due to rapid proteolysis. Additionally, while the gene sequence was adapted to *B. subtilis* codon usage, a certain incompatibility between the signal peptide and mature domain sequences of SP*Ka*POx and the *B. subtilis* secretory machinery has to be considered. *K. aureofaciens,* which belongs to the phylum Actinobacteria and is closely related to Streptomycetaceae [23], was previously classified as *Streptomyces aureofaciens* [24]. *B. subtilis* belongs to the Firmicutes, and differences between the Tat translocase complexes of *B. subtilis* (comprising two subunits, TatA and TatC) and those of *S. lividans* and other *Streptomyces* spp., which comprise three subunits (TatA, TatB and TatC), were reported [25,26]. It is conceivable that the native SP from *KaPOx* is not properly processed by the *B. subtilis* Tat system.

In bacteria, Auxiliary Activities Family 3 sequences are generally closely related to fungal POx sequences rather than to sequences from other subfamilies [6,27]. We have shown here that the pyranose oxidase from *K. aureofaciens* is very likely a secretory enzyme, which supports the discussed biological function as an Auxiliary Activity with biological roles in hydrogen peroxide provision and/or quinone redox cycling, as outlined in the Introduction. This raises the question whether more bacterial POx-like enzymes are secretory enzymes (and whether their annotations in genome data needs to be re-examined and perhaps revised). We performed a BLAST search (tblastn) using *Ka*POx as the query sequence, selected the 25 sequences with highest similarity and query coverage where upstream sequences were available, plus the characterized enzymes from *P. siccitolerans* and *S. canus*, and examined these upstream sequences for putative signal peptide sequences as described. The results are summarized in Table 1: 15 out of 27 sequences extended in frame upstream of the annotated start codon. In four cases, these additional sequences were less than twelve amino acids long. In two cases (both *Streptomyces* spp.), TatP-1.0 predicted the sequence to be a signal peptide with a score of 5 out of 5. One sequence (from *Actinoalloteichus* sp., of the family Pseudonocardiaceae) of 100 additional amino acids gave more than one additional sequence, one of which was classified as possibly constituting a signal peptide. The upstream sequence of the gene from *P. siccitolerans* was predicted with a score of 3 out of 5; two more sequences (from *Streptomyces* and an *Arthrobacter* species) resulted in a score of 2 out of 5. The sequences that contain putative upstream signal peptides are distributed across several branches of a phylogenetic tree constructed from extant bacterial POx-like sequences (Supplementary material Figure S4), and an allocation along taxonomic categories is not possible. It appears plausible that bacterial GMC-oxidoreductases, while generally similar to fungal POx sequences, have diversified to a range of biological functions, with some as secretory enzymes with an auxiliary activity in lignocellulose degradation, and others with a cytoplasmic location and a different biological role. Clearly these results are preliminary, and further investigations into the biochemical properties of bacterial POx-like sequences, their subcellular localization and biological function are necessary.

Most fungal POx enzymes have been purified from hyphal extracts [7,9,28,29], although Daniel et al. (1994) [30] proposed an extracellular localization based on microscopic studies. Nishimura et al. (1996) [31] expressed a cDNA from *Trametes* (*Coriolus*) *versicolor* in *E. coli* and reported that the first 38 amino acids were missing in the translated protein. Hallberg et al. [32] reported that the N-terminal part of *Trametes ochracea* (*multicolor*) pyranose oxidase has an unordered conformation and is not resolvable in the crystal structure [32]. These two enzymes show 96% identity, and both their N-terminal parts show similarities to a (bacterial) Tat signal peptide as analyzed by computational tools (TatP-1.0; Supplementary Material Figure S3). Pyranose oxidase has long been discussed as having been acquired by fungi from bacteria via Horizontal Gene Transfer (HGT) [27]. The fact that most fungal POx do not appear to be secretory enzymes (or that the Tat-SP-like sequence at the N-terminus of the *T. ochracea* and *T. versicolor* POx is not functional in fungi) does not necessarily contradict this—most lignocellulose-degrading fungi possess other enzymes for these biological functions. It is possible that different bacterial pyranose oxidase sequences were acquired by fungi on separate occasions and have diversified post-HGT for other roles. 

## 4. Materials and Methods

### 4.1. Strains and Plasmids

*Escherichia coli* JM109 and MC1061 (MoBiTec GmbH, Göttingen, Germany) were used as an intermediate cloning host. *Bacillus subtilis* NZ8901 (MoBiTec GmbH) and *Streptomyces lividans* TK24 were used as an expression host. Vector pUC19 was used for cloning purposes in *E. coli*, pNZ8901 was used as an expression vector in *B. subtilis* and pIJ486 was used as a backbone for expression in *S. lividans*. *S. lividans* TK24 and pIJ486 were a gift from Dr. Mohamed Belal Hamed (KU Leuven) [33,34]. 

### 4.2. Media

Standard Luria Bertani (LB) medium containing Ampicillin (100 µg/mL) or Chloramphenicol (10 µg/mL) was used for *E. coli* cultivation. Cultivation of *B. subtilis* was conducted in 2 × YT medium (per liter: 16 g tryptone, 10 g yeast extract, 5 g NaCl) containing chloramphenicol (5 µg/mL) as described in the supplier’s manual. *S. lividans* was cultivated as described [35] in phage medium (per liter: 0.5 g MgSO_4_·7H_2_O, 0.74 g CaCl_2_·2H_2_O, 10 g glucose, 5 g tryptone, 5 g yeast extract, 5 g Lab Lemco powder; the pH was adjusted to 7.2 with 5 N NaOH). For *S. lividans* transformation, R2 medium was used (per liter: 103 g sucrose, 0.25 g K_2_SO_4_, 12.12 g MgCl_2_·6H_2_O, 0.1 g casamino acids, 1 g yeast extract, 5 g of Lab Lemco powder, 100 mL TES buffer, 2 mL trace element solution, 10 mL 0.5% KH_2_PO_4,_ and 2% agar. 10 mL filter-sterile 36.8% CaCl_2_·2H_2_O and 1 mL filter-sterile 2 mM CuSO_4_ solution were added after sterilization). The trace element solution contained (per liter) 40 mg ZnCl_2_, 200 mg FeCl_3_⋅6H_2_O, 10 mg CuCl_2_⋅2H_2_O, 10 mg MnCl_2_⋅4H_2_O, 10 mg Na_2_B_4_O_7_⋅ 10H_2_O, and 10 mg (NH_4_)6Mo_7_O_24_⋅4H_2_O) and was filter-sterilized. Thiostrepton was used as a selective marker and added to a final concentration of 50 µg/mL from a stock solution of 50 mg/mL in DMSO. Chemicals and media components were purchased from Sigma Aldrich (St. Louis, MO, USA).

### 4.3. DNA Manipulation

Genomic extraction was carried out using Monarch^®^ Genomic DNA Purification Kit, and plasmid DNA was isolated using Monarch^®^ Plasmid Miniprep Kit. Polymerase Chain Reaction (PCR) was carried out using Q5^®^ High-Fidelity DNA polymerase with the oligonucleotides listed in Supplementary Table S1. Monarch^®^ PCR & DNA Cleanup Kit and Monarch^®^ DNA Gel Extraction Kit were used to purify PCR products. All extraction and purification kits and all DNA-modifying enzymes and restriction endonuclease were purchased from New England Biolabs (Ipswich, MA, USA).

### 4.4. Vector Constructions

Constructions based on the shuttle vector pNZ8901 for the *B. subtilis* expression system were done by amplifying the gene of interest from the genome of *K. aureofaciens* through standard PCR. Primers pNZ_SP*Ka*POx_F and *Ka*POxHis_R were used to amplify *spkapox* (the *Ka*POx gene harboring the putative signal peptide sequence and a C-terminal His-tag), while primers pNZ_*Ka*POx_F and *Ka*POxHis_R were used to amplify *kapox* (the *Ka*POx gene with a C-terminal His-tag). Restriction endonucleases *Pst*I and *Xba*I were used to digest both PCR products (*spkapox* and *kapox*) and pNZ8901 prior to ligation using T4 Ligase. The ligated products were transformed to *E. coli* MC1061 for propagation and transformed into *B. subtilis* NZ8901 as circular plasmids isolated from *E. coli* MC1061. 

Vector construction for *S. lividans* TK24 expression system was conducted in several steps due to the lack of a promoter in the pIJ486 backbone. The constitutive promoter (P_vsi_) from the subtilisin inhibitor gene (*vsi*) was amplified using primers P_vsi__F and P_vsi__R from pIJ486-*sp^sec^*-*mRFP* (gift from KU Leuven), digested by *Hind*III and *Pst*I and ligated to pUC19 digested with the same restriction endonucleases to generate pUC19-P_vsi_. Both primer pairs SP*Ka*POx_F with *Ka*POxHis_R and *Ka*POx_F with *Ka*POxHis_R were used to amplify *spkapox* and *kapox,* respectively, from the *K. aureofaciens* genome. Restriction endonucleases *Pst*I and *Xba*I were used to digest both PCR products and pUC19-P_vsi_ prior to ligation to generate pUC19-P_vsi_-*spkapox* and pUC19-P_vsi_-*kapox*. Constructs with the reporter gene *mrfp* were generated through Gibson Assembly^®^ (New England Biolab). We modified the pIJ486-*sp^sec^*-*mrfp* into pIJ486-*mrfp* by removing the signal peptide sequence, *sp^sec^*. Primer mRFP_GA_F (carrying an overhang of the P_vsi_ 3′ end sequence) and mRFP_GA_R (carrying an overhang of the pUC19 sequence) were used to amplify *mrfp* from pIJ486-*sp^sec^*-*mrfp*. Vector pUC19-P_vsi_ was linearized with *Pst*I, and Gibson Assembly^®^ was used to insert the amplification product to generate pUC19-P_vsi_-*mrfp*. The primer pair (SP)*Ka*POx_GA_F and *Ka*POx_GA_R was used to generate the PCR products (*sp)kapox* with an overhang of the P_vsi_ sequence on its 5′ end, a linker and an overhang with the mRFP sequence in its 3′ end. The primer pair mRFP_lkk_GA_F and mRFPHis_GA_R was used to generate *mrfp* with a linker on its 5′ end and a His-tag with a complementary sequence to pUC19 on its 3′ end. The PCR products (*sp*)*kapox-linker* and *linker-mrfp-his*, together with pUC-P_vsi_ linearized with *Pst*I, were used as a template for the Gibson Assembly^®^ reaction, to generate pUC19-P_vsi_-(*sp*)*kapox-mrfp*. All intermediate constructs in pUC19-P_vsi_ were digested with restriction enzymes *Hind*III and *Xba*I and ligated into pIJ486 digested with the same restriction enzymes. All constructs used in this study were listed in Table 2.

### 4.5. Transformation

Chemically competent cells were used to conduct transformation in *E. coli* [36], and electroporation was used for transformation in *B. subtilis* following the instructions in the supplier´s manual (MoBiTec GmbH). Transformation of *S. lividans* TK24 was carried out through protoplast preparation and transformation [35], using thiostrepton (50 µg/mL) as a selection marker.

### 4.6. Cultivation and Protein Production

*B. subtilis* harboring recombinant vector pNZ8901 and its derivatives were grown in 50 mL 2xYT medium containing chloramphenicol 5 µg/mL at 37 °C, 200 rpm. Recombinant protein production was induced by adding 1% (*v*/*v*) subtilin preparation when OD_600_ reached 0.8. The subtilin preparation was produced from *B. subtilis* NZ8963 (MoBiTec GmbH) according to the supplier´s manual. Cultures were harvested at 24 h and 48 h after induction. Then, 50 mL culture was centrifuged and the supernatant separated from the pellet and stored with the addition of protease inhibitor (10 µg/mL phenyl methyl sulfonyl fluoride; PMSF). The pellet was washed three times with phosphate buffer pH 7 and suspended in 5 mL buffer following the last washing step. PMSF was added at 10 µg/mL, and the cells were pre-treated with lysozyme (0.5 mg/mL for one hour at 37 °C) and disrupted by sonication on ice using a Bandelin Sonopuls HD 60 (Bandelin electronic GmbH, Berlin, DE) set at 80 V and 30%-cycle for 3 × 1 min, with two-minute intervals. Recombinant His-tagged protein was purified on 1 mL Ni-NTA columns (Merck, Darmstadt, Germany) following the manufacturers recommendations using Buffer A (50 mM Tris-HCl pH 7.5, 30 mM NaCl, and 30 mM Imidazole) as binding and washing buffer and Buffer B (50 mM Tris-HCl pH 7.5, 30 mM NaCl, and 250 mM Imidazole) as elution buffer.

Cultivation of *S. lividans* TK24 was conducted in baffled flasks containing 50 mL phage medium with 10 µg/mL thiostrepton at 30 °C, 150 rpm. No induction was required due to the use of the constitutive P_vsi_ promoter. Samples of 1 mL were obtained every 24 h and centrifuged (4000× *g*, 10 min, 4 °C). Supernatants were separated and stored at 4 °C with 10 µg/mL PMSF. Wet cell weight of the pellets was determined, and samples were washed three times with 1 mL phosphate buffer pH 7 containing 25 mM NaCl. Treatment with lysozyme and sonication was done as described for *B. subtilis* (sonication was done for only 1 min). The lysed samples were centrifuged at 13,000 rpm for 5 min at 4 °C, and extracts were stored with 10 µg/mL PMSF.

### 4.7. SDS-PAGE and Western Blot 

Samples were mixed with 2x SDS buffer mix (Sigma), denatured at 95 °C for 3 min and separated on Mini-PROTEAN^®^TGX Stain-Free™ Precast Gels 4–20% (Bio-Rad, Hercules, CA, USA) at 150 volt for 50 min. Precision Plus Protein^™^ Western C™ (Bio-Rad) was used as a molecular weight standard. Proteins were transferred to a 0.2 µm Nitrocellulose membrane by dry-blotting in a Trans-Blot^®^ Turbo™ (Bio-Rad) at 1.3 A, and 25 V for 7 min. Proteins were detected using a BSA-free anti-Penta-His-tag mouse monoclonal IgG (Qiagen, Hilden, Germany) as primary antibody and Polyclonal Rabbit-anti-mouse Immunoglobulin/HRP (Agilent, Santa Clara, CA, USA) as secondary antibody according to the manufacturer´s recommendations. Clarity Western ECL (Bio-Rad) was used as substrate. Visualization of both stain-free SDS-PAGE gel and Western blot was done in a ChemiDoc™ XRS+ (BioRad).

### 4.8. Enzymatic Activity Assay and Fluorescent Signal Analysis

To determine the *Ka*POx dehydrogenase activity, an enzymatic activity assay was performed using 160 mM glucose as electron donor and ferrocenium hexafluorophosphate 0.16 mM as electron acceptor. For the oxidase activity, atmospheric oxygen was used as electron acceptor. Quantification of oxidase activity was done using 1 mM 2,2′-Azino-bis (3-ethylbenzothiazoline-6-sulfonic acid; ABTS) and Horseradish Peroxidase (Sigma Aldrich; 143 U/mL). Dehydrogenase activity was determined spectrophotometrically at 300 nm, while oxidase activity was determined at 420 nm as described previously [6].

Detection of fluorescence of mRFP was conducted by exciting the samples at 584 nm and analyzing the emission at 603 nm. Both *Ka*POx enzymatic activities as well as mRFP fluorescent signals were measured in an EnSpire^®^ multimode plate reader (PerkinElmer, Waltham, MA, USA).

## Figures and Tables

**Table 1:** Prediction of putative Tat signal peptide using TatP-1.0.

No.	Species	E Value	Per. Ident	Upstream	Length (AA)	TatP-1.0	Note
1	*Kitasatospora aureofaciens*	0	95.24	v	44	Predicted	This study
2	*Mycobacterium lacus*	0	65.19	n.d			
3	*Actinomyces* sp. Lu 9419	6.00 × 10^−175^	56.75	v	101	n.d	
4	*Amycolatopsis japonica*	3.00 × 10^−168^	55.29	n.d			
5	*Frankia alni* ACN14a	7.00 × 10^−152^	51.06	n.d			
6	*Streptomyces sudanensis*	9.00 × 10^−140^	49.01	n.d			
7	*Streptomyces davawennsis* JCM 4913	3.00 × 10^−135^	48.99	v	55	n.d	
8	*Streptomyces alboniger*	8.00 × 10^−139^	48.91	v	56	Predicted	TatP-1.0 score 5 out of 5
9	*Streptomyces* sp. MRC013	1.00 × 10^−138^	48.74	n.d			
10	*Phytohabitans suffuscus*	4.00 × 10^−102^	48.15	n.d		n.d	
11	*Streptomyces cinnabarinus*	2.00 × 10^−135^	48.08	v	12	n.d	
12	*Streptomyces cyaneogriseus* subsp. *noncyanogenus*	2.00 × 10^−93^	48.04	v	52	n.d	TatP-1.0 score 2 out of 5
13	*Streptomyces huasconensis*	4.00 × 10^−136^	47.76	v	64	Predicted	TatP-1.0 score 5 out of 5
14	*Streptomyces tuirus*	6.00 × 10^−143^	46.94	v	32	n.d	
15	*Streptomyces lusitanus*	3.00 × 10^−139^	46.48	n.d			
16	*Phytohabitans flavus*	3.00 × 10^−125^	46.23	n.d			
17	*Nocardiopsis* sp. Mg02	4.00 × 10^−106^	38.95	v	10	n.d	
19	*Microbacterium* sp. 10M-3C3	5.00 × 10^−63^	38.25	v	50	n.d	
20	*Microbacterium atlanticum*	2.00 × 10^−60^	37.59	v	41	n.d	
21	*Microbacterium* sp. KUDC0405	1.00 × 10^−48^	37.59	v	5	n.d	
22	*Microbacterium testaceum* StLB037	6.00 × 10^−62^	37.55	n.d			
23	*Micromonospora carbonacea*	7.00 × 10^−76^	37.1	n.d			
24	*Arthrobacter* sp. DNA4	6.00 × 10^−59^	37.08	v	35	n.d	TatP-1.0 score 2 out of 5
25	*Actinoalloteichus* sp. AHMU CJ021	1.00 × 10^−59^	37.06	v	100	Predicted	More than 1 possibility for TatP-1.0 prediction
26	*Microbacterium* sp. XT11	7.00 × 10^−63^	37.02	v	10	n.d	
27	*Streptomyces canus*	9.00 × 10^−61^	33.71	n.d			
28	*Pseudarthrobacter siccitolerans*	6.00 × 10^−64^	34.89	v	60	Predicted	TatP-1.0 score 3 out of 5

**Table 2:** Vectors used in this study.

**Name**	**Recombinant Gene**	**Host**	**Origin**
pUC19	-	*E. coli*	NEB
pNZ8901	-	*E. coli/B. subtilis*	MoBiTec
pNZ8901-*spkapox*	*Ka*POx with signal peptide (*SPKaPOx*)	*E. coli/B. subtilis*	This study
pNZ8901-*kapox*	*KaPOx*	*E. coli/B. subtilis*	This study
pIJ486	-	*S. lividans* TK24	*KU Leuven*
pIJ486-*sp^sec^*-*mrfp*	*mRFP* with signal peptide	*S. lividans* TK24	*KU Leuven*
pUC19-P**_vsi_**	Promoter VSI (P_vsi_)	*E. coli*	This study
pUC19-P**_vsi_**-*mrfp*	P_vsi_, *mrfp*	*E. coli*	This study
pUC19-P**_vsi_**-*spkapox*	P_vsi_, *SPKaPOx*	*E. coli*	This study
pUC19-P**_vsi_**-*kapox*	P_vsi_, *KaPOx*	*E. coli*	This study
pUC19-P**_vsi_***_-_spkapox-mrfp*	P_vsi_, *SPKaPOx*, *mrfp*	*E. coli*	This study
pUC19-P**_vsi_***_-_kapox-mrfp*	P_vsi_, *KaPOx*, *mrfp*	*E. coli*	This study
pIJ486-*mrfp*	P_vsi_, *mrfp*	*S. lividans* TK24	This study
pIJ486-sp*kapox*	P_vsi_, *SPKaPOx*	*S. lividans* TK24	This study
pIJ486-*kapox*	P_vsi_, *KaPOx*	*S. lividans* TK24	This study
pIJ486-sp*kapox-mrfp*	P_vsi_, *SPKaPOx*, *mrfp*	*S. lividans* TK24	This study
pIJ486-*kapox-mrfp*	P_vsi_, *KaPOx*, *mrfp*	*S. lividans* TK24	This study

## Data Availability

Not applicable.

## Supplementary Materials

The supporting information can be downloaded at: https://www.mdpi.com/article/10.3390/ijms24031975/s1.
