# Supplementary material for: Localization of Pyranose 2-Oxidase from Kitasatospora aureofaciens: A Step Closer to Elucidate a Biological Role
Source: Int J Mol Sci. 2023 Jan 19;24(3):1975. doi: 10.3390/ijms24031975 (PMC9916811; doi:10.3390/ijms24031975)
Supplement: Supplementary file 1 [file ijms-24-01975-s001.zip › ijms-2147131-supplementary.pdf]

Supplementary Material

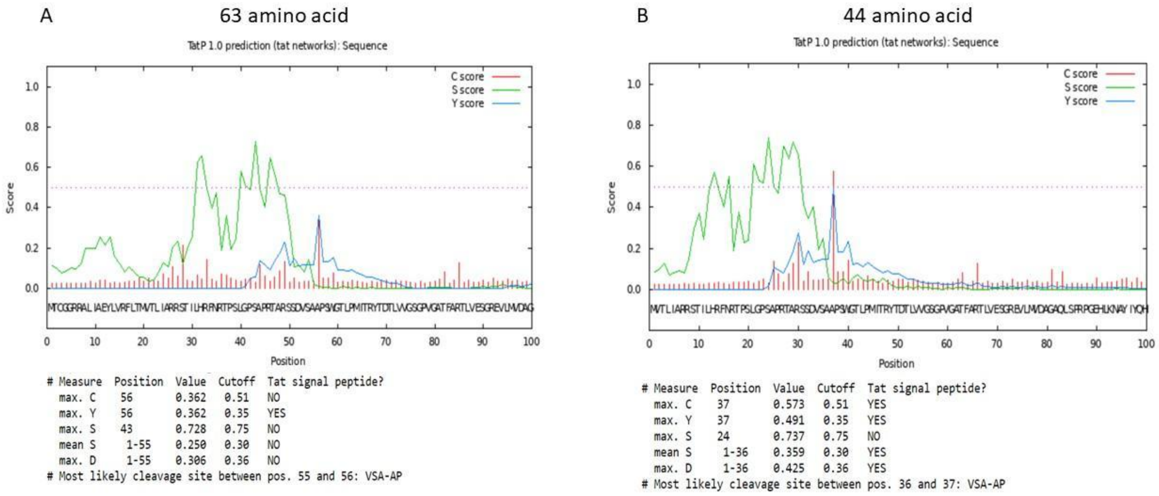

Figure S1: Signal peptide prediction using TatP-1.0 for the additional upstream 63 amino acids (A) and 44 amino acids (B)

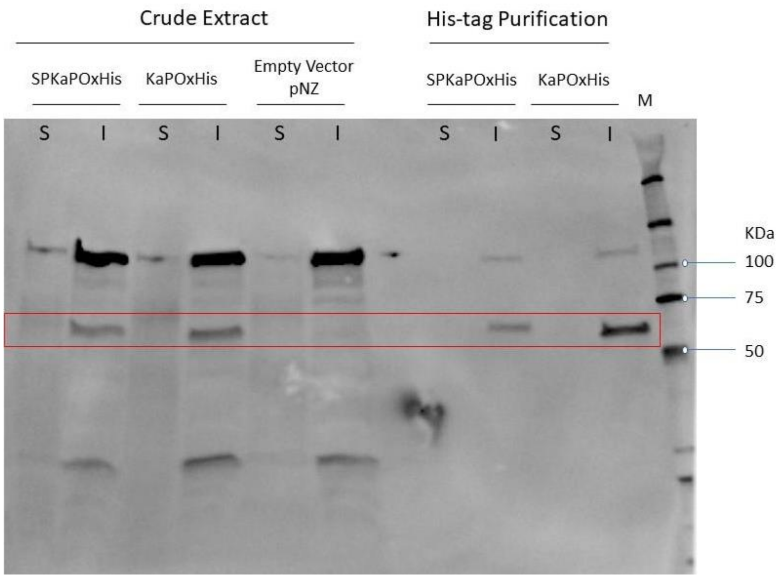

Figure S2: Western blot of samples from *B. subtilis* expression system harbouring SPKaPOxHis, KaPOxHis, and the empty pNZ vector in un-purified form (crude extract) and purified form (His-tag purification). Samples from supernatant are indicated as S, samples from the cell extract are indicated as I, the western blot marker is marked as M. Expected molecular weight of targeted protein was 60 KDa (shown in red box).

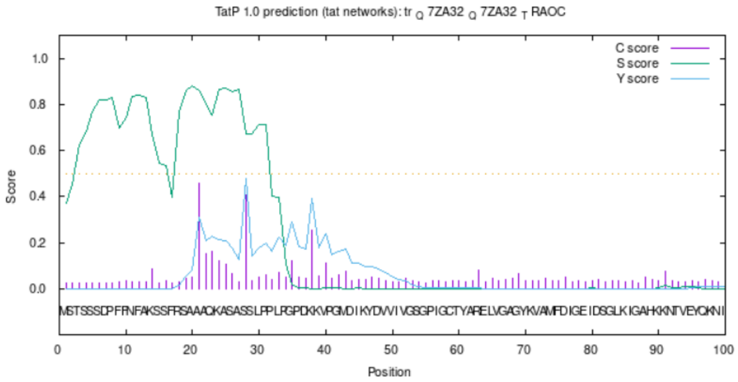

data

```
>tr_Q7ZA32_Q7ZA32_TRA  length = 100
# Measure  Position  Value  Cutoff  Tat signal peptide?
  max. C    21       0.456   0.51   NO
  max. Y    28       0.480   0.35   YES
  max. S    20       0.883   0.75   YES
  mean S     1-27    0.735   0.30   YES
  max. D     1-27    0.607   0.36   YES
# Most likely cleavage site between pos. 27 and 28: ASA-SS
# Potential Tat signal peptide but No Tat motif was found
# Used regex: RR.[FGAVML][LITMVF]
//
```

Figure S3: Signal peptide prediction using TatP-1.0 for Pyranose Oxidase from *Trametes ochracea* (POx Q7ZA32)

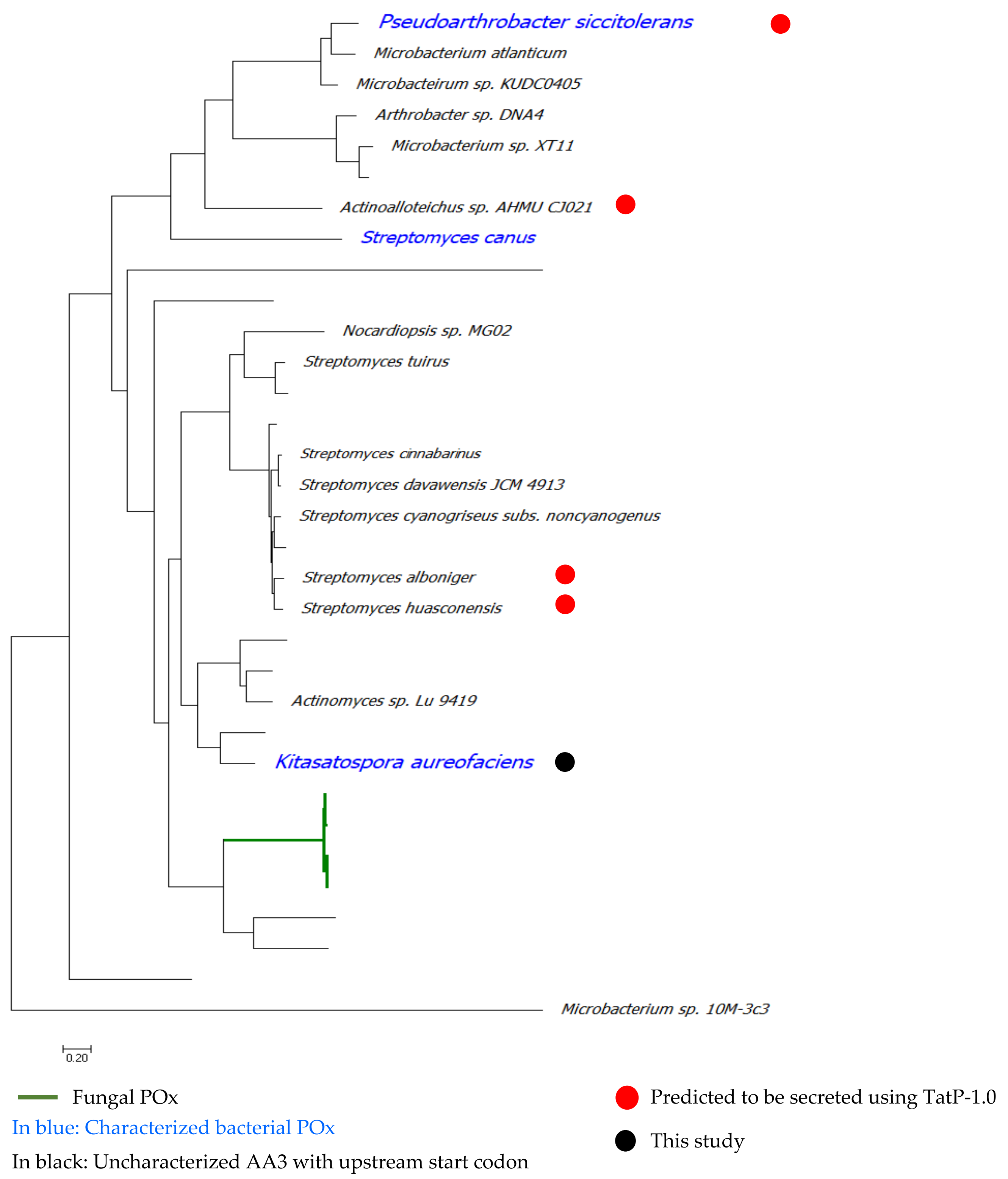

Figure S4. Phylogenetic tree constructed from extant bacterial POx-like sequences with *Ka*POx as query. Multiple alignment was conducted using Mafft (https://mafft.cbrc.jp/) with iterative refinement method for < 200 sequence with global homology. Tree was constructed using NGPhylogeny (https://ngphylogeny.fr/) with TrimAl as alignment curation and PhyML for tree inference.

Table S1. Oligonucleotides used in this study

Name	Sequence (5′ - 3′)	Note
pNZ_SPKaPOx_F	AACTGCAGAAATGGTTACATTGATAGCCCGTAGATCAAC	SPKaPOX to pNZ8901
pNZ_KaPOx_F	AACTGCAGAAATGATTACTCGCTATACGGAC	KaPOX to pNZ8901
KaPOxHis_R	CTCTCTAGATCAGTGATGATGATGATGATGGCTTCGGACAGCGAGCACCTC	(SP)KaPOX to pNZ8901 / pUC19-P_{vsi}
Pvsi_F	TTTTTTAAGCTTGGGGATGACCACCGCGGGAG	P_{vsi} to pUC19
Pvsi_R	TTTTTCATCTGCAGCTCTCCTTCGATCGATG	P_{vsi} to pUC19
SPKaPOx_F	GCTGCAGATGGTTACATTGATAGCCCG	SPKaPOX to pUC19-P_{vsi}
KaPOx_F	GAGAGCTGCAGATGATTACTCGCTATACGGACACC	KaPOX to pUC19-P_{vsi}
mRFP_GA_F	*CATCGATCGAAGGAGAG*CTGCAGATGGCCTCCTCCGAGGACGTC	mRFP to pUC19-P_{vsi} without SP^{Sec} with Gibson Assembly
mRFP_GA_R	*AGCTCGGTACCCGGGGATCC*GAGCTCTCTAGATTAGGCGCCGGTGGAGTGGCGGC	mRFP to pUC19-P_{vsi} without SP^{Sec} with Gibson Assembly
SPKaPOx_GA_F	CGATCGAAGGAGAGCTGCAGATGGTTACATTGATAGCCCGTAG	fusion SPKaPOx-mRFP-His in pUC19-P_{vsi}
KaPOx_GA_F	GCTTCGGACAGCGAGCACCTC	fusion KaPOx-mRFP-His in pUC19-P_{vsi}
KaPOX_GA_R	*CGGGGATCCGAGCTCTCTAGA***CCGCCGCTGCCGCC**GCTTCGGACAGCGAGCACCTCG	fusion (SP) KaPOx-linker in pUC19-P_{vsi}
mRFP_F	ATGGCCTCCTCCGAGGACGTCATCAAG	
mRFP_lkk_GA_F	**AGCGGCGGCAGCGGCGGT**ATGGCCTCCTCCGAGGACGTC	fusion (SP) KaPOx-mRFP-His in pUC19-P_{vsi}
mRFPHis_GA_R	*GGGGATCCGAGCTCTCTAGATCA*GTGATGATGATGATGATGGGCGCCGGTGGAGTGGCGCC	fusion (SP) KaPOx-mRFP-His in pUC19-P_{vsi}
eGFP_F	AGAGCTGCAGATGGTGAGCAAGGGCGAGGAG	eGFP to pUC-P_{vsi}
eGFP_R	GAGCTCTCTAGATCATTACTTGTACAGCTCGTCCATGCCGAGAGTGATCCC	eGFP to pUC-P_{vsi}

eGFP_GA_F	*CGAGTTGGGGGACTTTGCCG*GTGAGCAAGGGCGAGGAGCT	SPeGFP to pUC-P_{vsi}
eGFP_GA_R	*GGGGATCCGAGCTCTCTAGA*TTACTTGTACAGCTCGTCCATGC	SPeGFP to pUC-P_{vsi}
eGFP_A	ATGTGTCAGCGGCGCCGAGTTGGGGGACTTTGCCGATGGTGAGCAAGGGCGAGGAG	SPKaPOx to eGFP
eGFP_B	TCACTTGGGCCCTCGGCGCCGCGAACGGCTCGGTCCAGTGATGTGTCAGCGGCGCCGAGT	SPKaPOx to eGFP
eGFP_C	TCAACAATTTTGCACAGGTTCAACCGCACTCCATCACTTGGGCCCTCGGCGCCGCG	SPKaPOx to eGFP
eGFP_D	GAGAG<u>CTGCAG</u>ATGGTTACATTGATAGCCCGTAGATCAACAATTTTGCACAGGTTC	SPKaPOx to eGFP

Bold: linker
Underline: Restriction enzyme
Italic: complement
